# Chemically Modified Interleukin-6 Aptamer Inhibits Development of Collagen-Induced Arthritis in Cynomolgus Monkeys

**DOI:** 10.1089/nat.2015.0567

**Published:** 2016-02-01

**Authors:** Masao Hirota, Ikuo Murakami, Yuichi Ishikawa, Tomoki Suzuki, Shun-ichiro Sumida, Shigeru Ibaragi, Hayato Kasai, Naoto Horai, Daniel W. Drolet, Shashi Gupta, Nebojsa Janjic, Daniel J. Schneider

**Affiliations:** ^1^ Otsuka Pharmaceutical Co., Ltd., Tokushima, Japan.; ^2^ Shin Nippon Biomedical Laboratories, Ltd., Drug Safety Research Laboratories, Kagoshima, Japan.; ^3^ SomaLogic, Inc., Boulder, Colorado.

## Abstract

Interleukin-6 (IL-6) is a potent mediator of inflammatory and immune responses, and a validated target for therapeutic intervention of inflammatory diseases. Previous studies have shown that SL1026, a slow off-rate modified aptamer (SOMAmer) antagonist of IL-6, neutralizes IL-6 signaling *in vitro*. In the present study, we show that SL1026 delays the onset and reduces the severity of rheumatoid symptoms in a collagen-induced arthritis model in cynomolgus monkeys. SL1026 (1 and 10 mg/kg), administered q.i.d., delayed the progression of arthritis and the concomitant increase in serum IL-6 levels compared to the untreated control group. Furthermore, SL1026 inhibited IL-6-induced STAT3 phosphorylation *ex vivo* in T lymphocytes from human blood and IL-6-induced C-reactive protein and serum amyloid A production in human primary hepatocytes. Importantly, SOMAmer treatment did not elicit an immune response, as evidenced by the absence of anti-SOMAmer antibodies in plasma of treated monkeys. These results demonstrate that SOMAmer antagonists of IL-6 may be attractive agents for the treatment of IL-6-mediated diseases, including rheumatoid arthritis.

## Introduction

Rheumatoid arthritis (RA) is an autoimmune inflammatory disease associated with persistent synovitis and progressive joint damage [1,2]. Although the causes of RA are not fully understood, proinflammatory cytokines, such as tumor necrosis factor-alpha, interleukin-1 (IL-1) and interleukin-6 (IL-6), are known to be involved in the progression of this disease [3–6]. Constitutive overproduction of IL-6 is observed in the synovial fluid, bone marrow, and serum of patients with RA [7–11]. IL-6 activity in synovial fluid is greater than in serum [8], indicating that IL-6 is generated from activated and/or inflamed cells in articular cavities and is subsequently released into serum. The abnormally high concentration of IL-6 exacerbates disease progression, and normalization of serum IL-6 levels is an effective treatment for this disease [12,13].

There is no cure for RA, and current treatments are designed to slow progression of the disease. First-line therapies for RA include nonsteroidal anti-inflammatory drugs and small-molecule disease-modifying antirheumatic agents such as methotrexate; however, there is a growing role for biological agents, including tocilizumab, a humanized anti-IL-6 receptor antibody [14] that blocks IL-6 signaling. Tocilizumab is an approved drug for treatment of RA and other diseases mediated by IL-6, such as Castleman's disease, juvenile idiopathic arthritis, and Crohn's disease [14–16].

IL-6 is a pleiotropic cytokine that regulates immune response, inflammation, hematopoiesis, and bone metabolism [17–20]. IL-6 activates cells by binding to its specific nonsignaling IL-6 receptor α (IL-6R, gp80, or CD126) present on the cell membrane. This ligand-receptor complex then binds to the signal-transducing protein gp130 (CD130) and activates the JAK-STAT3 (Janus kinase–signal transducers and activators of transcription 3) signaling pathway [21,22], resulting in STAT3 phosphorylation, a critical step in many IL-6 activities [23–25]. Soluble IL-6R (sIL-6R) in blood and other body fluids binds to IL-6, but instead of acting as an antagonist, sIL-6R increases the half-life of IL-6 and activates the signaling pathway in cells which do not express the membrane-bound IL-6Rα [26–28].

We previously reported the discovery and optimization of SL1025, a single-stranded DNA slow off-rate modified aptamer (SOMAmer) that binds with high affinity to human (K_d_ = 0.2 nM) and monkey (K_d_ = 2.5 nM) IL-6 and inhibits IL-6-dependent cell signaling pathways [29]. Similar to traditional aptamers, SOMAmers are selected *in vitro* from large random libraries, but are uniformly functionalized with hydrophobic moieties (eg, benzyl-, 2-naphthyl-, or 3-indolyl-carboxamide) at the 5-position of uridine through a carboxamide linker [30]. These hydrophobic groups can participate in interactions with target molecules as well as form novel intramolecular secondary and tertiary structural motifs [31,32]. In addition to improved affinities, which are comparable to those of antibodies, SOMAmer technology offers several advantages over traditional aptamers, including enhanced nuclease resistance and greater selection success rates [33].

**FIG. 1.**
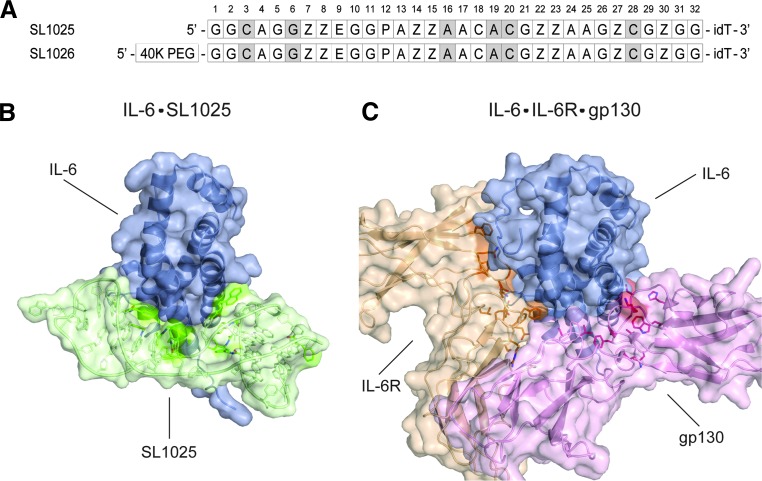
SL1025 occludes binding sites of IL-6R and gp130. **(A)** Sequences of SL1025 and SL1026 with 5-dU modifications indicated (Z = benzyl, *P* = naphthyl, E = phenethyl) and 2′-methoxy positions highlighted *gray*. SL1026 is comprised of SL1025 with a 40 kDa polyethylene glycol (PEG) conjugated to its 5′ terminus. Both sequences have a 3′ inverted dT (idT). **(B)** X-ray crystal structure of the IL-6:SL1025 complex in a cartoon and transparent surface rendering representation (PDB ID: 4NI9) [31]. IL-6 is colored *blue*, and SL1025 is colored *green*. Hydrophobic modifications in SL1025 that make direct contact with IL-6 are highlighted *green*. **(C)** X-ray crystal structure of the IL-6:IL-6R:gp130 complex in a cartoon and transparent surface rendering representation (PDB ID: 1P9M) [67]. IL-6 is colored *blue*, IL-6R is colored *brown,* and gp130 is colored *pink*.

SL1025 is a 32 nucleotide sequence with ten hydrophobic modifications (eight benzyl, one naphthylmethyl and one phenylethyl), as well as six 2′-methoxy ribose modifications to further enhance nuclease stability (Fig. 1A). Analysis of the crystal structure of SL1025 in a complex with IL-6 revealed that the majority of the IL-6 contact surfaces for both IL-6R and gp130 are occluded by SL1025 in the complex [31] (Fig. 1B, C). Furthermore, nearly all of the hydrophobic modifications are clustered on one side of SL1025 and make direct contact with IL-6.

SL1026 is a PEGylated form of SL1025 and has similar affinity (K_d_ = 0.2 nM) for human IL-6 and similar inhibition activity for human IL-6 [29] and monkey IL-6 (data not shown). In this study, we report that SL1026 can delay the progression of RA in a nonhuman primate collagen-induced arthritis (CIA) model. We also show that SL1026 can inhibit STAT3 phosphorylation in human T lymphocytes as well as C-reactive protein (CRP) and serum amyloid A (SAA) protein production in human primary hepatocytes. Taken together, these results show that SL1026 is a potent antagonist of the IL-6 signaling pathway and represents a potential new drug candidate for the treatment of IL-6-mediated diseases, including RA.

## Materials and Methods

### Reagents

SL1026 was prepared with a hexylamine modification at the 5′ terminus by solid phase synthesis at Agilent Technologies (Boulder, CO) as described previously [29]. Polyethylene glycol (PEG) (branched 2 × 20 kDa NHS ester; JenKem Technology, Plano, TX) was conjugated to the terminal amine using standard methods. SOMAmer concentrations for all studies were calculated using only the mass of the DNA component (excluding the mass of the PEG component). SL1026 for animal studies was tested for bacterial endotoxin contamination by the Limulus amebocyte lysate method [34] and determined to be below the lower limit of assay detection (<0.009 EU/mg). Human recombinant IL-6 was purchased from Millipore, Inc. (Billerica, MA). Tocilizumab (Actemra^®^ 200 mg) was manufactured by Genentech, Inc. (San Francisco, CA).

### Measurement of STAT3 inhibition

Measurement of STAT3 phosphorylation in T lymphocytes was conducted as described previously [35]. Whole blood was collected from the antecubital vein of 10 healthy Japanese human volunteers. SL1026 or tocilizumab was pre-equilibrated with human recombinant IL-6 (10 μg/mL, 0.4 μM) and incubated with 200 μL of human blood for 20 min at 37°C. After removing the red blood cells with fluorescence activated cell sorting (FACS) lysing solution (BD Biosciences, San Diego, CA), the cells were suspended in 1 mL of methanol to permeabilize the cell membrane. Methanol was removed and the cells were then resuspended in 100 μL of FACS buffer (phosphate buffered saline [PBS] containing 2% fetal bovine serum) containing 10% AlexaFluor 488-conjugated anti-p-STAT3 antibody (BD Biosciences), 0.2% PE-conjugated anti-CD3 antibody (BD Biosciences), and 0.2% PerCP-conjugated anti-CD4 antibody (BD Biosciences). After incubation for 1 h on ice, the cells were resuspended in 700 μL of FACS buffer. Samples were analyzed using a FACS Calibur Flow Cytometer (Becton Dickinson and Company, Tokyo, Japan), and the mean fluorescence intensity of the cells was analyzed using CellQuest software version 3.3 (Becton Dickinson and Company). Protocols were approved by the Ethics Committee of the Otsuka Pharmaceutical Company and conducted in accordance with the guidelines for human experimentation established by the Declaration of Helsinki. Each subject provided written informed consent to participate in the study.

### Measurement of CRP and SAA

Human primary hepatocytes (KAC Co., Ltd., Kyoto, Japan) were seeded into 96-well plates at a concentration of 2.8 × 10^4^ cells/well with incubation medium (KAC Co., LTD.). The following day, fresh medium was added containing human recombinant IL-6 (10 ng/mL) pre-equilibrated with SL1026 or tocilizumab. Twenty-four hours later, supernatants were collected and the concentration of CRP and SAA were determined by ELISA. CRP was measured with a CircuLex High-Sensitivity CRP ELISA Kit (CircuLex Co., Ltd., Nagano, Japan), and SAA was measured with an Invitrogen Hu SAA ELISA Kit (Thermo Fisher Scientific, Inc., Waltham, MA).

### Animal care

Twenty-four female cynomolgus monkeys (*Macaca fasciularis*), aged 3–5 years were obtained from Guangxi Grandforest Scientific Primate Company, Ltd. (Guangxi, China). Twelve monkeys were used for the pharmacokinetic study and 12 were used for the CIA model study. Animals were housed individually at a temperature of 26°C ± 3°C and relative humidity of 55 ± 20%. Monkey chow (HF Primate 5K91 12G 5K9J; Purina Mills, LLC) was provided at ∼108 g/day and tap water was provided *ad libitum* from an automatic supply system (Edstrom Industries, Inc., Waterford, WI). Studies were performed by Shin Nippon Biomedical Laboratories, Ltd. (Kagoshima, Japan) in accordance with standards published by the National Research Council (Guide for the Care and Use of Laboratory Animals, NIH OACU) of the National Institutes of Health Policy on Human Care and Use of Laboratory Animals. In accordance with these standards, every effort was made to ensure that the animals were free of pain and discomfort.

### Pharmacokinetic study

SL1026 was formulated in a vehicle consisting of 10 mM phosphate buffer (pH 7) containing 5 mM MgCl_2_, 135 mM NaCl, and 0.05% (w/v) Polysorbate 20. SL1026 was administered by bolus injection into the cephalic vein. Twelve animals were assigned to 3 dose groups (*n* = 4 per group): 1, 10, and 30 mg/kg. Blood was collected from the femoral vein in K_2_EDTA vacutainers (BD Biosciences) at 0.083, 0.25, 0.5, 1, 2, 4, 6, 8, 12, 24, 48, and 72 h after dose administration. SL1026 concentrations in plasma were measured by the dual hybridization method [36]. Briefly, a capture probe with a 3′ amine was designed to hybridize with 17 bases on the 5′ terminus of SL1026, and a detection probe with a 5′ fluorescein isothiocyanate (FITC) label was designed to hybridize with 15 bases on the 3′ terminus of SL1026. The capture probe was immobilized in a 96-well activated plate (Sumitomo Bakelite, Tokyo, Japan) and the plate was washed and blocked. The detection probe was incubated with the plasma samples for 15 min at 80°C to allow annealing to SL1026. Plasma samples were then added to the capture probe plate and incubated for 2 h at 38°C to allow annealing to SL1026. After washing the plate, horseradish peroxidase (HRP)-conjugated anti-FITC antibody (Southern Biotechnology Associates, Inc., Birmingham, AL) was added to each well and incubated for 2 h at room temperature. After washing the plate, HRP substrate solution (0.5 mg/mL 3,3′,5,5′-tetramethylbenzidine [TMB], 0.33 mM EDTA-2K, 0.2% acetic acid, 25% diethylformamide) was added to each well and incubated for 7–15 min at room temperature. Reactions were stopped by the addition of 0.5 M H_2_SO_4_ and absorbance was measured. Blank and standard samples were prepared for each plate and a calibration curve was used to determine the SL1026 concentration in each plasma sample. Pharmacokinetic parameters were determined by noncompartmental analyses using WinNonlin software (version 5.2.1; Pharsight Corp., St. Louis, MO).

### CIA study

Twelve cynomolgus monkeys were assigned to 3 groups (4 animals per group): control and two SL1026-treated groups (1 and 10 mg/kg/dose). Arthritis was induced by collagen treatment as described previously [37]. Bovine type II collagen (K41S type2 collagen, 0.4% solution) was purchased from Collagen Research Center (Tokyo, Japan), diluted to 4 mg/mL and then mixed with an equal volume of complete Freund′s adjuvant (BD Biosciences). Monkeys were immunized on Study Day 0 by intradermal 2 mL injections into the back. Animals received a booster 3 weeks later (day 21) by the same procedure. SL1026 was formulated as described above and intravenous bolus doses (1 mL/kg) were administered into the cephalic vein every 6 h for 11 days starting on the first day of immunization. The control group received vehicle alone following the same dosing schedule. Arthritis scores and general condition scores of monkeys were observed and recorded once a week for 5 weeks, at 6, 12, 20, 27, and 34 days after the first immunization. Clinical assessment was performed according to established methods, which were modified in consideration of joint function [37]. Arthritis scores were evaluated by monitoring the degree of swelling and rigidity at the metacarpophalangeal, proximal interphalangeal, and distal interphalangeal joints, and of the wrist, ankle, elbow, and knee (total 64 joints). Each joint was assessed according to the following evaluation criteria: Score 0, no abnormality; Score 1, swelling not visible, but can be determined by touch; Score 2, swelling slightly visible and can be confirmed by touch; Score 3, swelling clearly visible, but joint can be completely flexed; Score 4, swelling clearly visible, but joint cannot be completely flexed; Score 5, rigid joints. The arthritis score for each animal was designated as the total score of individual joints. General condition scores were evaluated by monitoring behavior and movement of the monkeys. Each monkey was assessed according to the following evaluation criteria: Score 0, No abnormality; Score 1, Difficulty in hanging from the bars of the home cage by the fingers; Score 2, Inability to hang from the bars of the home cage by the fingers (using wrist); Score 3, Movement only by using forelimbs or hindlimbs; Score 4, Crouching; Score 5, Abnormal body position. Arthritis and general condition scores were determined by investigators blind to treatment assignment.

### Measurement of SL1026, IL-6, and anti-SL1026 antibody in monkey plasma

Blood was drawn from the femoral vein of each monkey. Samples were processed into plasma using K_2_EDTA for the measurement of SL1026 concentration, or using heparin sodium (Ajinomoto Pharmaceutical Co. Ltd., Tokyo, Japan) for the measurement of IL-6 and anti-SL1026 antibody. SL1026 concentration in plasma was measured by the dual hybridization method as described above. IL-6 concentration in the plasma was measured using a Quantikine human IL-6 ELISA Kit (R&D Systems, Inc., Minneapolis, MN). SL1026 did not interfere with the measurement of IL-6 with this kit (data not shown). Anti-SL1026 antibody in the plasma was measured by ELISA. Briefly, an immunoplate (Sumitomo Bakelite) was coated with 50 pmol/well of the DNA component or with 10 pmol/well of the PEG component of SL1026 according to the manufacturer's instructions. Monkey plasma (1,000-fold diluted) was added to each well and incubated for 2 h at room temperature with shaking (200 rpm). After washing the wells with PBS containing 0.05% Polysorbate, horseradish peroxidase-conjugated anti-human IgG+IgM+IgA (H&L) (Biodesign, Saco, ME) was diluted 40,000-fold in PBS containing Polysorbate and added to the wells for 1 h at room temperature with shaking (200 rpm). After washing, TMB substrate (Thermo Fisher Scientific) was added, and the colorimetric reaction was measured using an EMax plate reader (Molecular Devices, Tokyo, Japan) at 450 nm. Normal cynomolgus monkey plasma served as a negative control, while an anti-DNA antibody (Millipore) and an anti-PEG antibody (Epitomics, Burlingame, CA) served as positive controls.

### Statistics

Graphical presentations and calculations were carried out using Microsoft Excel 2003 (SP1; Microsoft Co., Redmond, WA). Statistical analyses were performed using SAS System for windows (release 9.1 and 9.3; SAS Institute, Inc., Cary, NC). For the *ex vivo* test using human lymphocytes, the Dunnett's test and unpaired *t*-test were conducted. For the monkey studies, the mixed effect model for repeated measures method was used for the comparison of clinical scores. Two-tailed *P* < 0.05 was considered significant. For the comparison of IL-6 concentrations on day 34, the Kruskal–Wallis test was performed comparing treatment groups with control, followed by a Dunn's post test corrected for multiplicity of comparison.

## Results

### Inhibition of IL-6-induced STAT3 phosphorylation in human T lymphocytes

**FIG. 2.**
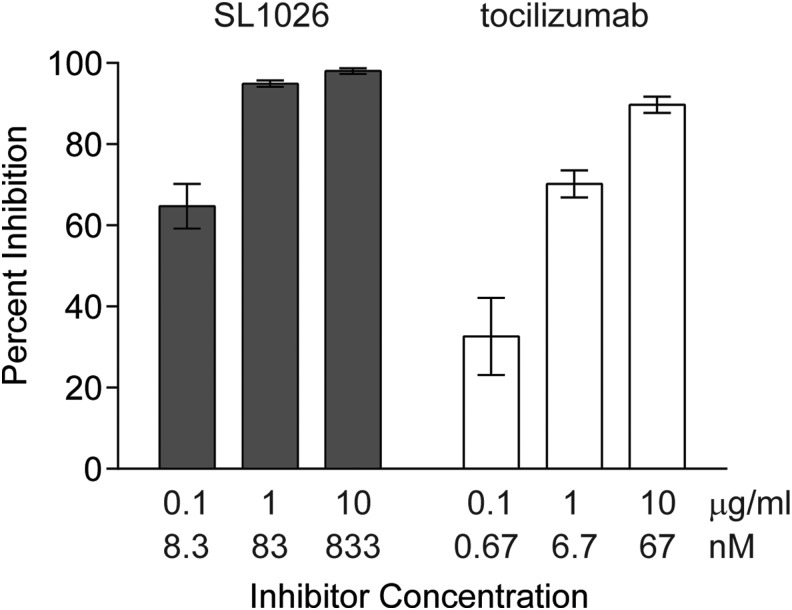
SL1026 inhibits IL-6-induced STAT3 phosphorylation in human lymphocytes. Cells were induced with IL-6 and STAT3 phosphorylation was determined by FACS using a fluorescent anti-p-STAT3 antibody. Percent inhibition values (relative to no IL-6 and no inhibitor control samples) are plotted as the mean ± SEM of 10 samples at each concentration. A statistically significant increase of inhibition was observed compared to no-inhibitor control (0.0 ± 2.8%) for all SL1026 and tocilizumab groups (Dunnett's test, two-tailed, *P* < 0.01).

To evaluate the inhibitory effect of SL1026 on IL-6-induced phosphorylation of STAT3, we performed an *ex vivo* assay using human peripheral blood lymphocytes (Fig. 2). IL-6 treatment led to a 7.5-fold increase in STAT3 phosphorylation of CD3^+^ and CD4^+^ lymphocytes, compared to the vehicle control. Treatment of cells with 0.1, 1, or 10 μg/mL of SL1026 (8.3, 83, or 833 nM) inhibited STAT3 phosphorylation 64.7 ± 5.5%, 94.7 ± 0.8%, and 98.0 ± 0.7% (mean ± SD), respectively. For comparison, 0.1, 1, or 10 μg/mL of tocilizumab (0.67, 6.7, or 67 nM) inhibited STAT3 phosphorylation 32.6 ± 9.5%, 70.2 ± 3.3%, and 89.7 ± 2.0%, respectively.

### Inhibition of IL-6-induced CRP and SAA production in human primary hepatocytes

**FIG. 3.**
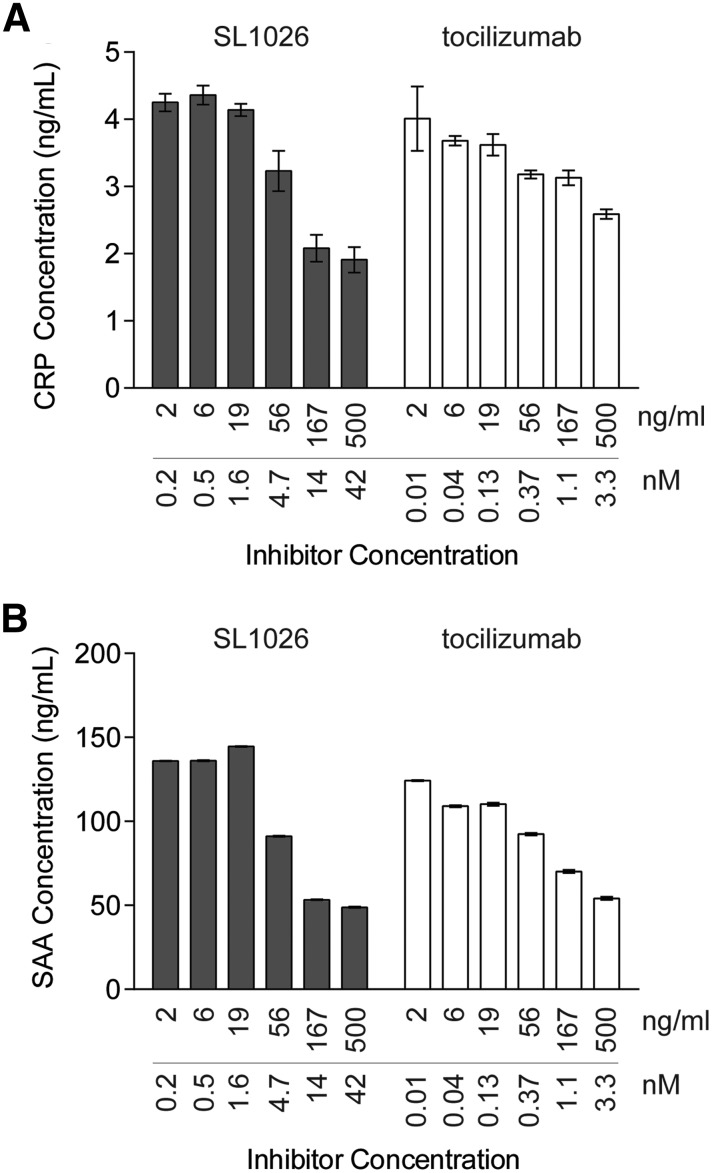
SL1026 inhibits IL-6-induced C-reactive protein (CRP) and serum amyloid A (SAA) expression in human primary hepatocytes. CRP **(A)** and SAA **(B)** concentrations were measured in hepatocyte supernatants by ELISA after IL-6 treatment and plotted as a function of SL1026 or tocilizumab concentration. Values are plotted as the mean ± SEM of three determinations.

To evaluate the inhibitory effect of SL1026 on IL-6-induced production of CRP and SAA, we performed an *ex vivo* assay using human primary hepatocytes (Fig. 3). CRP and SAA concentrations in supernatants from hepatocytes were 1.8 and 9.4 ng/mL (mean, *n* = 3), respectively. Treatment of cells with IL-6 increased CRP concentration ∼2-fold and SAA more than 10-fold compared to nonstimulated cells. Similar to tocilizumab, SL1026 showed dose-dependent inhibition of IL-6-induced production of CRP and SAA.

### Pharmacokinetics of SL1026

**FIG. 4.**
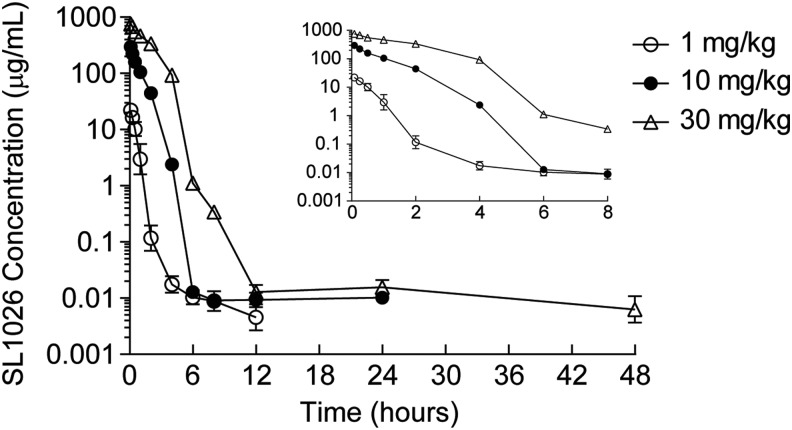
Plasma concentration–time profiles of SL1026 in monkeys. Plasma SL1026 concentrations after administrations of 1 (○), 10 (●), or 30 mg/kg (△) were measured. Values are plotted as the mean ± SD of four monkeys. Values measured during the first 8 h after administration are shown in the *inset* plot.

To establish a dose regimen for the CIA study, a plasma pharmacokinetic evaluation was performed in female cynomolgus monkeys following a single 1, 10, or 30 mg/kg intravenous bolus dose (*n* = 4 per group). Mean extrapolated maximum SL1026 plasma concentrations (C_0_) were approximately dose linear over the 30-fold dose range studied (Table 1). Plasma concentration–time curves showed a biphasic decline (Fig. 4) with mean terminal (t½ ß) half-lives of 5.33, 164, and 51.8 h for the 1, 10, and 30 mg/kg dose groups, respectively (Table 1). As assessed by plasma area under the concentration-time curves (AUC), the increase of total SL1026 exposure with dose was greater than dose-proportional, indicating saturation of plasma clearance. Thus, over the dose range studied, plasma clearance values declined 4-fold from 79.1 mL/h/kg for the low-dose group to 19.9 mL/h/kg for the high-dose group.

**Table 1.** Mean Pharmacokinetic Parameters of SL1026 After Single Bolus Administration in Cynomolgus Monkeys

	*Dose (mg/kg)*		
*Pharmacokinetic parameter*	*1*	*10*	*30*
C_0_ (μg/mL)	25.8	359	803
t_1/2_ (h)			
α	0.359	0.387	0.912
β	5.33	164	51.8
AUC_t_ (h·μg/mL)	13.5	309	1,520
AUC_inf_ (h·μg/mL)	13.6	306	1,520
CL (mL/h/kg)	79.1	32.7	19.9
V_z_ (mL/kg)	611	7,770	1,510
V_ss_ (mL/kg)	39.2	117	30.9

Values were calculated from three or four monkeys in each group.

C_0,_ initial blood level; t_1/2,_ half-life; α, half-life of the first phase; β, half-life of the second phase; AUC_t_, area under the concentration–time curve from time 0 to the last measurable time point; AUC_inf_, area under the plasma concentration–time curve from time 0 to infinity; CL, clearance; V_z_, apparent volume of distribution during the terminal phase; V_ss_, apparent volume of distribution at steady state.

### Reduction of arthritis symptoms in monkeys treated with SL1026

**FIG. 5.**
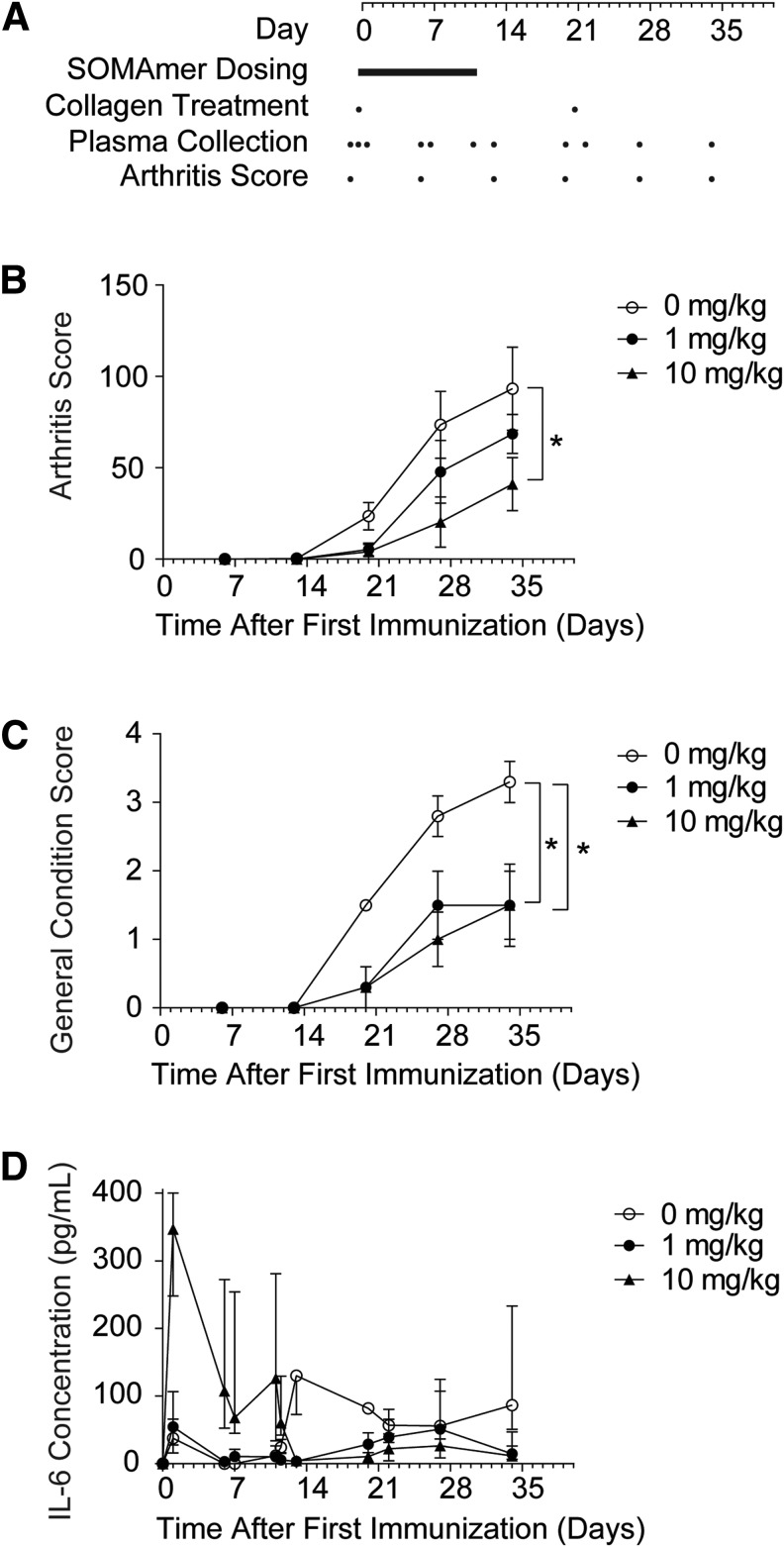
Effect of SL1026 on plasma IL-6 levels and clinical assessments in collagen-induced arthritis (CIA) monkeys. **(A)** Animals were sensitized on day 0 and 21 with collagen and dosed with slow off-rate modified aptamer (SOMAmer) every 6 h on days 0–11. Plasma was collected and measurements of arthritis score and general condition score were made on days indicated with a *dot*. Arthritis score **(B)** and general condition score **(C)** were evaluated at days 6, 13, 20, 27, and 34 after the first sensitization. Values are plotted as the mean ± SEM (*n* = 4 in each group) for groups administered 0 (○), 1 (●), or 10 (▲) mg/kg SL1026. A statistically significant decrease of arthritis score was noted in the 10 mg/kg group (*P < 0.05, 10 mg/kg group vs. 0 mg/kg group) and of general condition score in both the 1 and 10 mg/kg groups (*P < 0.05, 1 and 10 mg/kg groups vs. 0 mg/kg group) by mixed effect model for repeated measures method in overall mean. **(D)** IL-6 concentration was measured in plasma samples collected at various times after the first immunization. Values are plotted as the median and interquartile range (*n* = 4 in each group) for groups administered 0 (○), 1 (●), or 10 (▲) mg/kg SL1026.

Monkeys received a q.i.d. administration of either 0, 1, or 10 mg/kg SL1026 (0, 4, or 40 mg/kg/day), with the schedule of collagen treatment, plasma collection, and arthritis score assessment indicated in Fig. 5A. All monkeys in the untreated control group (0 mg/kg SL1026) began to show clinical signs of arthritis on day 13, with an arthritis score of 0.3 ± 0.3 (mean ± SEM). This score continued to increase throughout the study and reached 93.3 ± 22.8 on day 34 (Fig. 5B). In contrast, no clinical signs of arthritis were detected until day 20 in monkeys treated with either 1 or 10 mg/kg SL1026. On day 34, arthritis scores for SL1026-treated animals were 68.5 ± 10.7 and 41.0 ± 14.6 for the 1 and 10 mg/kg dose groups, respectively. The reduced arthritis score of the 10 mg/kg dose group on day 34 was significantly different than the control group (*P* < 0.05). The general condition score for the untreated control monkeys also continued to worsen throughout the study, reaching a value of 3.3 ± 0.5 (mean ± SEM) on day 34 (Fig. 5C), compared to 1.5 ± 0.5 and 1.5 ± 0.6 for the 1 and 10 mg/kg SL1026 treatment groups, respectively. The improved general condition score for both treatment groups on day 34 was significantly different than the control groups (*P* < 0.05). Overall, SL1026 dose-dependent improvements in both arthritis score and general condition score were observed in this study.

### Plasma SL1026 concentration in monkeys with CIA

Plasma SL1026 concentrations were measured throughout the CIA study and are shown in Table 2. The concentrations of SL1026 5 min after the first 1 or 10 mg/kg dose were 19.4 and 184 μg/mL (1.6 and 15.4 μM), respectively, reflecting the dose-dependent initial exposure on day 0. Peak plasma concentrations measured on days 7 and 11 were similar to those on day 0. To determine the trough plasma concentrations, plasma samples were collected immediately before the first SL1026 administration of day 1 (5th dose) and day 6 (25th dose). SL1026 trough concentrations in the 10 mg/kg group were significantly greater than predicted by the single-dose concentration–time profile. In both the 1 and 10 mg/kg groups, the mean plasma trough concentrations on day 6 were 2–3 times greater than on day 1 (0.018 μg/mL and 0.049 μg/mL on day 1 and day 6, respectively for the 1 mg/kg group, and 12.7 μg/mL and 26.6 μg/mL on day 1 and day 6, respectively for the 10 mg/kg group). Furthermore, SL1026 was still detectable in the plasma at 0.011 μg/mL (1 mg/kg group) and 0.082 μg/mL (10 mg/kg group) on day 13, ∼48 h after the final administration.

**Table 2.** Mean Peak and Trough SL1026 Plasma Concentrations After the First Dose Administration of the Indicated Study Day in Cynomolgus Monkeys with Collagen-Induced Arthritis

	*SL1026 concentration (μg/mL)*					
	*Day 0*	*Day 1*	*Day 6*	*Day 7*	*Day 11*	*Day 13*
1 mg/kg						
Peak^a^	19.4	—	—	19.7	19.1	—
Trough^b^	—	0.018	0.049	—	—	—
48 h after last dose	—	—	—	—	—	0.011
10 mg/kg						
Peak^a^	184	—	—	172	164	—
Trough^b^	—	12.7	26.6	—	—	—
48 h after last dose	—	—	—	—	—	0.082

^a^ Sample collected 5 min after first dose on indicated day.

^b^ Sample collected immediately before first dose on indicated day.

Mean values are reported (*n* = 4).

### Plasma IL-6 concentration in SL1026-treated monkeys

In control monkeys, median plasma concentrations of IL-6 remained at or below 37.8 pg/mL throughout the dosing period (days 0–11), but rapidly increased to 130 pg/mL on day 13 and then remained at or above 56.0 pg/mL through day 34 (Fig. 5D). In monkeys administered 10 mg/kg SL1026, median IL-6 concentrations dramatically increased to 347 pg/mL on day 1, but then steadily decreased to 4.7 pg/mL by day 13 where median values remained at or below 26.4 pg/mL through day 34. In monkeys administered 1 mg/kg SL1026, median IL-6 concentrations were similar to the control group during the treatment period and showed only a moderate increase from days 13–34, peaking at 51.1 pg/mL on day 27. On the final day of the study (day 34), the difference between median IL-6 concentrations in the SL1026 treatment groups was significantly different than the control group (*P* = 0.0263). After dosing, a trend toward reduced IL-6 concentrations was observed in SL1026-treated animals compared to control animals (*P* = 0.079 for 1 mg/kg SL1026 vs. control and *P* = 0.037 for 10 mg/kg SL1026 vs. control).

### Plasma anti-SL1026 antibody titers in monkeys treated with SL1026

**FIG. 6.**
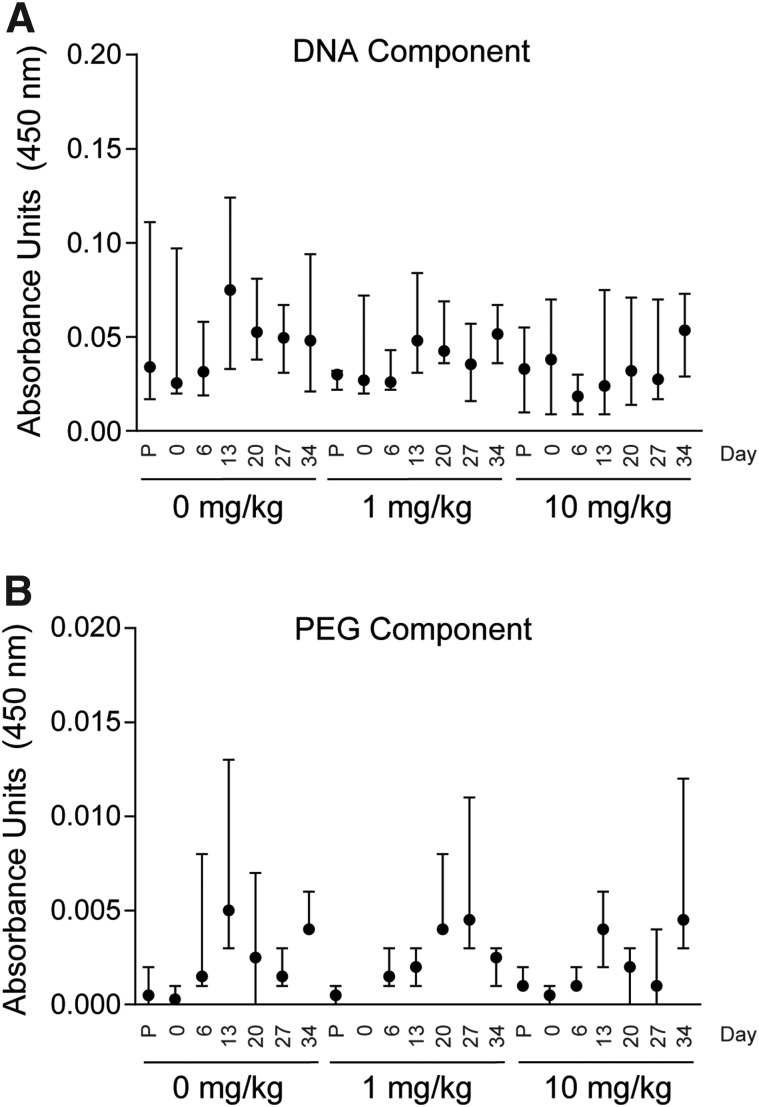
Anti-SL1026 antibody levels in CIA monkeys. Antibodies against the DNA component of SL1026 **(A)** and the PEG component of SL1026 **(B)** were measured in plasma collected from all monkeys 6 days before the first SL1026 dose (P), and on days 0, 6, 13, 20, 27, and 34 after the first SL1026 dose. Values are plotted as median and range (*n* = 4 in each group).

We screened for production of antibodies against the DNA and PEG components of SL1026. Compared to predose, no anti-SL1026 antibodies were detected for the DNA or PEG components of SL1026 over the 34-day experimental period (Fig. 6). The coating of the DNA and PEG components was confirmed with anti-DNA and anti-PEG antibodies. No signal was observed in this assay with normal monkey plasma (data not shown).

## Discussion

The nonhuman primate CIA model is an established system for studying RA [37], and the therapeutic and preventive effects of several existing drugs, including tocilizumab, have been assessed in this model [38]. Animals display an autoimmune-mediated polyarthritis, synovitis, and erosion of cartilage and bone [37,39,40]. These symptoms begin to manifest at the clinical onset of arthritis [41], and are similar to human RA [42]. As in human RA, IL-6 is believed to be one of the important triggers in this monkey model, and is thought to play a key role in contributing to the severity of disease [43]. SL1026 treatment delayed the onset of arthritis in monkeys in this study and reduced the severity of symptoms. Not only did joint swelling and stiffness occur at a lower frequency in monkeys treated with SL1026 compared to the control monkeys, but also an overall improvement in general health condition was observed. Furthermore, SOMAmer treatment resulted in a sustained reduction in plasma IL-6 levels that corresponded precisely with the reduction in RA symptoms.

SL1026 administration began on the day animals received their first collagen immunization. Thus, SL1026 was given before the expected increase in serum IL-6, allowing SL1026 to access target tissues before the onset of inflammation. Results from the single-dose pharmacokinetic study in monkeys indicated an expected plasma concentration of 13 ng/mL (1 nM) at 6 h after administration of a 10 mg/kg dose (Fig. 4). Based on these results, a dosing regimen of four administrations per day was chosen to ensure a plasma concentration of SL1026 in excess of the *in vitro* IC_50_ value (2.4 nM) [29] for nearly the entire dosing interval for the 10 mg/kg dose. SL1026 measurements in samples collected during the study (Table 2) indicated that SL1026 concentrations remained above the IL-6 concentration (less than 500 pg/mL, Fig. 5D) at both dosing concentrations.

The trough concentrations of SL1026 were 0.018 μg/mL and 0.049 μg/mL in plasma collected 5 min before the 5th and 25th dose at 1 mg/kg, and 12.7 μg/mL and 26.6 μg/mL in plasma collected 5 min before the 5th and 25th dose at 10 mg/kg, ∼1,000-fold greater than predicted by the single-dose concentration–time profile. This suggests that the clearance rate was decreasing after repeated doses, perhaps due to saturation of a clearance mechanism, allowing measurable concentrations of SL1026 (11 and 82 ng/mL in the low- and high-dose groups, respectively) to remain in the plasma on day 13, ∼48 h after the final administration. Even if SL1026 concentrations dropped below the presumed pharmacologically effective level in plasma after the last administration, we expected SL1026 to accumulate in target tissues, such as articular cavities, by the enhanced permeation and retention effect [44–46]. Thus, pharmacologically effective concentrations of SL1026 might have persisted at the target tissues long after the final dose and contributed to the overall inhibition of the onset of arthritis in joints and restoration of the general health condition of the treated animals.

Serum IL-6 levels reflect the normal endogenous production of IL-6 [47], and IL-6 is an important serum biomarker for treatments that exert their effects through IL-6 signal inhibition. The rate of IL-6 clearance from the blood is increased significantly upon binding IL-6R [48], and anti-IL-6R antibodies such as tocilizumab block this clearance mechanism, leading to a temporary increase in free IL-6 levels in serum of animals and humans [47,48]. While tocilizumab significantly reduces the disease activity of RA by effectively inhibiting IL-6 signal transduction and the subsequent anti-inflammatory response [49,50], the increase in blood levels of free IL-6 may result in high IL-6 exposure to organs during drug treatment.

Horai *et al.* reported serum IL-6 concentrations in monkeys when CIA begin to rise at about 14 days after the first immunization and peak at 21–28 days [37]. This observation was recapitulated in our control group, but in the treatment groups, an increase in total serum IL-6 was observed during the drug administration period, particularly at the high dose (Fig. 5D). IL-6 concentrations returned to baseline in the treatment groups after administration of the final dose on day 11, and were consistently lower than in the control group from days 13–34 during the expected peak period. Notably, arthritis scores and general health scores in the treatment groups were lower than those in the control group during this same period. IL-6 levels in the 1 mg/kg dose group were only slightly greater than the control group during SL1026 dosing, but remained lower than the control group after dosing, providing an intermediate reduction in RA symptoms.

Because SL1026 is known to block binding of IL-6 to IL-6R, the IL-6 spike during SOMAmer administration may have been due to interference of receptor-dependent IL-6 elimination, as was observed with tocilizumab. Additionally, the rise in IL-6 levels during SOMAmer administration may be an indication of an on-target effect commonly seen with antibody drugs, where inhibitor binding alters the rates of target distribution and elimination, resulting in increased plasma target concentrations. [51–53]. However, while total IL-6 levels increased during SL1026 administration, free IL-6 levels likely decreased, as the majority of serum IL-6 existed as a complex with SOMAmer and was, therefore, inactive (IL-6 was not detected in plasma after depletion of SL1026:IL-6 complexes with anti-PEG antibody-coated beads, data not shown). This inhibition of IL-6 activity during the early stages of disease development led to the reduction in RA symptoms in both dose groups. We worried that the rise in IL-6 levels during SOMAmer dosing could be due to activation of toll-like receptors (TLR) by SL1026, or contaminating endotoxin. However, no evidence of TLR9 activation was observed in an *in vitro* cellular assay with as much as 230 μM SL1026 (data not shown), and the endotoxin level in the test material was below the detectable measurement limit (<0.009 EU/mg).

Activation of CD4^+^ lymphocytes has been observed in the earliest clinical stage of RA [54], and IL-6 induction of STAT3 phosphorylation in T lymphocytes is believed to be closely associated with RA [55–57]. SL1026 fully inhibited IL-6 signaling and STAT3 phosphorylation in isolated human T lymphocytes with potency comparable to tocilizumab. Additionally, IL-6 induces production of CRP and SAA by hepatocytes, and tocilizumab was previously shown to inhibit this activity by blocking the IL-6 signal transduction pathway [19]. Similar to tocilizumab, SL1026 exhibited dose-dependent inhibition of IL-6-induced production of CRP and SAA by isolated human primary hepatocytes. These *ex vivo* results further support the *in vivo* observations and indicate that the suppression of RA symptoms in the CIA model resulted directly or indirectly from IL-6 signal inhibition by SL1026.

Due to the clinical success of tocilizumab, IL-6 signal blockade is considered to be a powerful therapeutic strategy for the treatment of RA. Many other biologics targeting IL-6 are in development [58,59], including other anti-IL-6R antibodies (such as sarilumab [60]), anti-IL-6 antibodies (such as sirukumab [61], siltuximab [62], clazakizumab [63], and olokizumab), and anti-gp130 antibodies and their fusion proteins [64]. All of these agents are antibodies or synthesized proteins, while SL1026 is a nucleic acid-based antagonist with certain advantages over antibody drugs *in vivo*. Antibody drugs can induce an immune response in patients after several administrations, whereby neutralizing antibodies are generated against the drug and generally weaken its efficacy [38,65,66]. This has not been observed for aptamer therapies to date, and in this study, anti-SL1026 antibodies were not detectable in any of the monkeys during the examination period. Furthermore, the aggressive dosing regimen of up to 10 mg/kg of SL1026 every 6 h for 11 days was well tolerated in all animals and no adverse effects were observed.

IL-6 is a multifunctional cytokine that promotes cell growth and differentiation and influences the expression of a variety of proteins. In addition to its role in inflammation, IL-6 is known to regulate tumor development, including initiation, promotion, malignant conversion, invasion, and metastasis, and a relationship between IL-6 expression and cancer pathology has been reported [67]. SL1026 was previously shown to inhibit the growth of several tumor cell lines *in vitro* [29], and has the potential to be an effective suppressor of tumor proliferation *in vivo*. These combined studies confirm that SL1026 is a potent antagonist of the IL-6 signaling pathway and represents a new class of drug candidate for the treatment of IL-6-mediated diseases including RA, inflammation, and cancer.
